# Neuroimmunology of Human T-Lymphotropic Virus Type 1-Associated Myelopathy/Tropical Spastic Paraparesis

**DOI:** 10.3389/fmicb.2019.00885

**Published:** 2019-04-24

**Authors:** Satoshi Nozuma, Steven Jacobson

**Affiliations:** Viral Immunology Section, Division of Neuroimmunology and Neurovirology, National Institute of Neurological Disorders and Stroke, National Institutes of Health, Bethesda, MD, United States

**Keywords:** human T-lymphotropic virus type 1, HTLV-1-associated myelopathy/tropical spastic paraparesis, neurological disorders, immunology, pathogenesis

## Abstract

Human T-lymphotropic virus type 1 (HTLV-1) is the etiologic agent of both adult T-cell leukemia/lymphoma and HTLV-1-associated myelopathy/tropical spastic paraparesis (HAM/TSP). HAM/TSP is clinically characterized by chronic progressive spastic paraparesis, urinary incontinence, and mild sensory disturbance. Given its well-characterized clinical presentation and pathophysiology, which is similar to the progressive forms of multiple sclerosis (MS), HAM/TSP is an ideal system to better understand other neuroimmunological disorders such as MS. Since the discovery of HAM/TSP, large numbers of clinical, virological, molecular, and immunological studies have been published. The host-virus interaction and host immune response play an important role for the development with HAM/TSP. HTLV-1-infected circulating T-cells invade the central nervous system (CNS) and cause an immunopathogenic response against virus and possibly components of the CNS. Neural damage and subsequent degeneration can cause severe disability in patients with HAM/TSP. Little progress has been made in the discovery of objective biomarkers for grading stages and predicting progression of disease and the development of molecular targeted therapy based on the underlying pathological mechanisms. We review the recent understanding of immunopathological mechanism of HAM/TSP and discuss the unmet need for research on this disease.

## Introduction

Human T-lymphotropic virus type 1 (HTLV-1) is the first human retrovirus discovered and infects 10–20 million people worldwide (Poiesz et al., 1980; de The and Bomford, 1993). Highly endemic areas include Southern Japan, the Caribbean, South America, Central and Southern Africa, Middle East, and Central Australia. Although the majority of infected individuals remain lifelong asymptomatic carriers (ACs), approximately 2–5% develop adult T-cell leukemia/lymphoma (ATLL) (Uchiyama et al., 1977) and another 0.25–3.8% develop HTLV-1-associated myelopathy/tropical spastic paraparesis (HAM/TSP) (Gessain et al., 1985; Osame et al., 1986). HTLV-1 has also been associated with other chronic inflammatory diseases including uveitis, myositis, dermatitis, and pulmonary infiltrative pneumonitis (Gessain and Mahieux, 2012). HTLV-1 can be transmitted through sexual contact, intravenous drug use, and breastfeeding from mother to child. While ATLL is mainly associated with breastfeeding, HAM/TSP can manifest in infected individuals by all routes of transmission (Yamano and Sato, 2012). The mean age of onset is 40–50 years, and the frequency is higher in women than in men (Gessain and Mahieux, 2012). The interaction between the host immune system and HTLV-1-infected cells regulates the development of HAM/TSP. In particular, HTLV-1-specific CD8-positive cytotoxic T lymphocytes (CTL) against HTLV-1 have been thought to play a pivotal role in the development of HAM/TSP (Matsuura et al., 2010; Saito, 2014). Two HTLV-1 viral regulatory proteins, Tax and HTLV-1 basic leucine zipper factor (HBZ), have been demonstrated to be involved in HTLV-1 infectivity and proliferation (Matsuoka and Jeang, 2011). Tax have been intensely studied because Tax induces the expression of many cellular genes and consequently contribute to cell activation and proliferation observed in HAM/TSP. HBZ has been recently recognized to play a critical role in inflammation and pathogenesis of HAM/TSP (Enose-Akahata et al., 2017). Although HAM/TSP is not directly life-threatening, the disease severely impacts patients’ quality of life (Olindo et al., 2006; Coler-Reilly et al., 2016), and there is no satisfactory curative treatment. Here, we review our recent understanding of the immunopathogenesis and the clinical features of HAM/TSP and discuss the need for continued basic and clinical research.

### Human T-Lymphotropic Virus Type 1

HTLV-1 belongs to the *Deltaretrovirus* genus of the *Orthoretrovirinae* subfamily of retroviruses. HTLV-1 integrates a single copy of the provirus into the genome of the host cell (Cook et al., 2012). HTLV-1 proviral genome contains structural genes, *gag*, *pol*, and *env* flanked by long terminal repeat at the both the 5′ and 3′ ends. HTLV-1 genome also has a *pX* region encoded several accessory genes including *tax*, *rex, p12*, *p21*, *p30*, *p13*, and *HTLV-1 basic leucine zipper factor* (*HBZ*) (Matsuoka and Jeang, 2007). The viral genes are transcribed from the 5′LTR, but only *HBZ* encoded on the minus strand of the provirus is transcribed from the 3′LTR. Two of these accessory genes, *tax* and *hbz*, play a key role in HTLV-1 pathogenesis. HTLV-1 Tax is a transcriptional transactivator of virus replication and induces the expression of a variety of cellular genes by activation of the NF-κB and CREB/ATF pathways (Matsuoka and Jeang, 2011). Tax is associated with many of the characteristic immune abnormalities observed in HAM/TSP and is related to dysfunction in immune cells of HAM/TSP patients (Enose-Akahata et al., 2017). *Ex vivo*, Tax protein is spontaneously expressed in peripheral blood mononuclear cells (PBMCs) after culture without any exogenous stimulation (Hanon et al., 2000a), and the level of *tax* mRNA was significantly higher in HAM/TSP patients than in ACs (Yamano et al., 2002). Tax is an immunodominant antigen recognized by HTLV-1-specific cytotoxic CD8^+^ T-cells (CTLs) (Jacobson et al., 1990). The number of Tax-specific CTLs is greatly elevated and these CTLs produce proinflammatory cytokines (Kubota et al., 1998) and show degranulation activity in HAM/TSP patients that is comparable to that in ACs (Abdelbary et al., 2011). Though Tax is usually undetected *in vivo*, recent analysis shows that Tax protein is expressed in intermittent but intense bursts at the single-cell level (Billman et al., 2017; Mahgoub et al., 2018). HBZ plays a key role in the growth and survival of leukemic cells; however, little is known about its role in the immunopathogenesis of HAM/TSP (Enose-Akahata et al., 2017). As HBZ closely cooperates with Tax, HBZ has opposing functions to Tax and modify transcription of various host genes (Matsuoka and Jeang, 2011). HBZ is persistently expressed in infected cells, maintains viral latency (Philip et al., 2014), and promotes proliferation of ATLL cells (Satou et al., 2006; Arnold et al., 2008). HBZ interacts with CREB/ATF pathway, suppresses Tax-mediated transactivation, and selectively inhibits the classical NF-κB pathway (Matsuoka and Jeang, 2011). The level of *HBZ* mRNA that was detected in HAM/TSP patients was significantly lower than that in ATLL patients but higher than in ACs. Furthermore, *HBZ* mRNA expression was associated with proviral load and increased disease severity in HAM/TSP patients (Saito et al., 2009). HBZ is also an immunogenic protein recognized by HBZ-specific CTL clones; however, HBZ is considered to be a weaker immunogen for CTLs then Tax. HBZ-specific CTL clones could not lyse ATLL cells (Suemori et al., 2009) and HBZ-specific CTL clones killed significantly fewer infected cells than were killed by Tax-specific CTL clones (Rowan et al., 2014). Antibody response against HBZ was detected in HTLV-1-infected subjects, but the antibody test could not distinguish between different clinical outcomes (Enose-Akahata et al., 2013). The lower immunogenicity of HBZ could allow HTLV-1-infected cells to escape from the host immune response.

HTLV-1 proviral load, which is strongly related to the risk of developing HAM/TSP, remains relatively stable within each subject while HTLV-1 drives a strong proliferation of infected T-cells (Bangham et al., 2015). The genomic location of the provirus is identical in every cell within an individual infected clone but differs between clones. Integration of HTLV-1 appeared to occur in genes associated with transcriptional start sites, and CpG island (Doi et al., 2005; Derse et al., 2007). Analysis of proviral integration sites between HTLV-1-infected individuals demonstrated that frequent integration into transcriptionally active sites was associated with an elevated rate of Tax expression (Meekings et al., 2008). Furthermore, a larger number of distinct HTLV-infected T-cell clones was detected in HAM/TSP patients than in ACs (Gillet et al., 2011). The frequency of spontaneous Tax expressing cells is considerably higher in clones of low abundance than in those of high abundance (Melamed et al., 2013). These results indicate that oligoclonal proliferation of HTLV-1-infected cells does not account for the development of HAM/TSP and clonal expansion of infected cells might be controlled by host immune response to Tax or by other viral factor such as HBZ in HAM/TSP patients.

### Current Topics: Extraordinary High Prevalence in Central Australia

One of the hot topics in HTLV-1 is the high prevalence in Central Australia, where more than 40% of Indigenous adults in some remote communities are HTLV-1c infected (Einsiedel et al., 2016b). HTLV-1 infection in the Australo-Melanesian region was observed in the early 1990s (Gessain and Cassar, 2012), but high prevalence rates in Central Australia has not been recognized until recently. As discussed below, HTLV-1c is one of the genetic subtypes of HTLV-1, which is found only in Oceania. HTLV-1 sequence in subtype c that infect the indigenous Australians reveals the high genetic diversity, while the sequence variability within subtype a, which is the most common worldwide, is very low (Cassar et al., 2013). High sequence diversity in HTLV-1c is considered to be due to a long period of evolution in isolated populations living on different islands of the Pacific area (Gessain and Cassar, 2012). Several studies have reported that common clinical manifestations of HTLV-1 infection in Indigenous Australians are bronchiectasis and blood stream infections, which are associated with higher HTLV-1c proviral load (Einsiedel et al., 2014a, 2016a). These findings are consistent with the proposed immunopathology of HTLV-1-related inflammatory diseases, in which the higher proviral load induces various immunologic abnormalities. Little is known about more common disease caused by HTLV-1 including HAM/TSP and ATLL, and only few cases have been described among the Indigenous population of Central Australia (Einsiedel et al., 2014b). Our knowledge of the global prevalence of HTLV-1 is still insufficient, and the risk of the retrovirus to the public health worldwide is underestimated. Future analysis is apparently needed for the clinical and basic research to clarify the pathogenetic, immunological and oncogenic mechanisms of this unknown variant of HTLV-1.

## Risk Factors

### Proviral Load

An elevated HTLV-1 proviral load (PVL) is the main risk factor for developing HAM/TSP in HTLV-1 infected subjects. The HTLV-1 PVL is measured as the number of HTLV-1 DNA copies per PBMCs and is usually expressed as the percentage of infected PBMCs. The PVL is 16-fold higher in HAM/TSP patients than in carriers and associated with an increased risk of progression to disease (Nagai et al., 1998). This high PVL is associated with multiple immunologic abnormalities that contribute to the development of HAM/TSP. When compared to ACs, HAM/TSP patients have higher levels of spontaneous lymphocyte proliferation (Sakai et al., 2001; Pinto et al., 2011), increased HTLV-1 expression, and importantly, a higher frequency of HTLV-1 specific CD8^+^ T-cells (Kubota et al., 2000; Yamano et al., 2002), which are thought to be critical mediators of central nervous system (CNS) injury. PVL is significantly elevated in cerebrospinal fluid (CSF) of HAM/TSP patients than in ACs and HTLV-1-infected patients with other neurological disorders (Puccioni-Sohler et al., 2007). Furthermore, higher PVL is also detected in CSF than in PBMCs of HAM/TSP (Nagai et al., 2001; Brunetto et al., 2014) and correlated with spinal cord atrophy (Azodi et al., 2017). The PVL of HTLV-1 reaches a stable level in each individual (Matsuzaki et al., 2001), which is maintained by the equilibrium between the proliferation of infected cells and their elimination by activated CTLs (Bangham, 2003). Although higher PVL is clearly associated with developing HAM/TSP, an elevated PVL alone is an incomplete requirement since a small minority of AC with a high proviral load can also develop HAM/TSP. Given that HTLV-1 PVLs are higher in ACs genetically related to HAM/TSP patients than in non-HAM/TSP-related ACs (Nagai et al., 1998), genetic factors might contribute to the replication of HTLV-1.

### Host Genetic Factors

Several host genetic factors, including human leukocyte antigen (HLA) and non-HLA gene polymorphisms affect the occurrence of HAM/TSP (Saito and Bangham, 2012). The HLA class I genotype determines the specificity and the efficacy of their CD8^+^ T-cell response and consequently has a large impact on the proviral load and the risk of developing HAM/TSP. Possession of the HLA-class I gene HLA-A*02 and Cw*08 was associated with a significant reduction in both HTLV-1 PVL and the risk of HAM/TSP (Jeffery et al., 1999, 2000; Catalan-Soares et al., 2009), while individuals with HLA-class I HLA-B*5401 and class II HLA-DRB1*0101 was indicated to have the susceptibility of developing with HAM/TSP (Jeffery et al., 1999, 2000). Analysis of non-HLA host genetic factors showed that single nucleotide polymorphisms (SNPs) in a small number of candidate genes affect the risk of developing HAM/TSP. Tumor necrosis factor (TNF)-α promoter −863 A (Vine et al., 2002) and the longer CA repeat alleles of matrix metalloproteinase (MMP)-9 promoter (Kodama et al., 2004) were associated with disease susceptibility, whereas interleukin (IL)-10 −592 A (Sabouri et al., 2004), stromal-derived factor-1 + 801 A, and IL-15 + 191°C alleles (Vine et al., 2002) were related to protective effect. IL6 promoter -634C allele frequencies were higher among HAM/TSP patients than among ACs in Brazil (Gadelha et al., 2008). IL28B gene SNP rs8099917 was reported to be associated with HAM/TSP in Brazil (Assone et al., 2014). Familial clusters of HAM/TSP were reported, but genetic analysis was not able to detect any disease-associated genes due to small number of cases (Nozuma et al., 2014, 2017). Comprehensive analysis of host genetics in a larger sample size and cases focusing on familial clustering might be useful to detect the susceptibility genes of HAM/TSP.

### HTLV-1 Genotype

HTLV-1 is segregated into seven major genetic subtypes (1a–1g) based on the nucleotide diversity of its LTR region, including a cosmopolitan subtype (subtype 1a), a Melanesian/Australian subtype (subtype 1c), and five African subtypes (subtype 1b, 1d, 1e, 1f, and 1g) (Verdonck et al., 2007). The cosmopolitan subtype is further divided into five sub-subtypes (Proietti et al., 2005), and globally the most common HTLV-1 genotype is transcontinental subtype (Gessain and Cassar, 2012). HTLV-1 has remarkably low genetic variability, although minor variations exist between geographical isolates (Komurian et al., 1991). Most studies of HTLV-1 genotype have reported no correlation between nucleotide substitutions and the risk of HAM/TSP (Mahieux et al., 1995), and the recent analysis of complete HTLV-1 sequence could not detect any HAM/TSP-specific mutations (Pessoa et al., 2014; Nozuma et al., 2017). However, the transcontinental HTLV-1 subtype is reported to be more frequently detected in HAM/TSP patients than in ACs in southern Japan, where the transcontinental and Japanese HTLV-1 subtypes co-exist (Furukawa et al., 2000; Nozuma et al., 2017). HAM/TSP patients with transcontinental subtype showed lower levels of *HBZ* mRNA expression (Yasuma et al., 2016) and higher levels of CXCL10, which has been proposed to be a prognostic biomarker for HAM/TSP (Naito et al., 2018). Different HTLV-1 subgroups revealed different patterns of host gene expression (Naito et al., 2018), which also might contribute to the higher incidence of HAM/TSP infected with transcontinental subtype.

## Host Immune Responese To HTLV-1

### Neuropathology

The main pathological feature in HAM/TSP is a chronic inflammation with diffuse degeneration throughout the central nervous system (Izumo et al., 2000). The spinal cord shows loss of myelin and axons symmetrically in the lateral and posterior column of the thoracic cord with the inflammation of grey and white matter, most notably at the thoracic level (Iwasaki, 1990; Yoshioka et al., 1993). These lesions are associated with perivascular and parenchymal lymphocytic infiltration with associated axonal loss, demyelination, reactive astrocytosis and fibrillary gliosis (Umehara et al., 1993). CD4^+^ and CD8^+^ cells were evenly distributed in active inflammatory lesions, while the predominance of CD8^+^ cells and high levels of interferon (IFN)-γ were observed in the chronic stage of disease (Umehara et al., 1993; Aye et al., 2000). A recent radiological study also demonstrated that a more atrophic spinal cord in HAM/TSP was associated with higher percentage of inflammatory CD8^+^ T-cells and HTLV-1 PVL in CSF (Azodi et al., 2017). HTLV-1-infected cells were determined to be CD4^+^ lymphocytes in the central nervous system (CNS) (Matsuoka et al., 1998), and HTLV-1 Tax-specific CTLs could be detected in the spinal cord (Matsuura et al., 2015). HTLV-1 has not been shown to actively infect neurons, oligodendrocytes, or microglia *in vivo* (Lepoutre et al., 2009). Alteration in tight junctions between endothelial cells in the vasculature causes blood-brain barrier (BBB) disruption with subsequent T-cell transmigration into the CNS in HAM/TSP (Afonso et al., 2007, 2008). Higher levels of MMP-2 and MMP-9 were detected in the CSF and in infiltrating perivascular mononuclear cells in active lesions in the CNS, indicating that MMP-2 and MMP-9 may play a key role in the BBB breakdown in HAM/TSP patients (Umehara et al., 1998). Cell adhesion molecules, such as intercellular adhesion molecule-1 (ICAM-1), vascular cell adhesion molecule 1 (VCAM-1), and cell adhesion molecule 1 (CADM1/TSLC1), and activated leukocyte cell adhesion molecule (ALCAM/CD166) have shown to be modulated by Tax (Valentin et al., 1997; Nejmeddine et al., 2009; Curis et al., 2016; Manivannan et al., 2016). Increased adhesion molecule expression might therefore facilitate the recruitment and migration of HTLV-1 infected lymphocytes through the BBB endothelium.

### Cytotoxic T Lymphocytes

The CD8^+^ cytotoxic T-cells (CTLs) response has been considered to play an important role in the development of HAM/TSP. One of the most prominent features of the cellular immune response in HAM/TSP patients is that the number of HTLV-1-specific CTLs is greatly elevated in PBMCs compared with ACs (Jacobson et al., 1990; Kubota et al., 2003). The immunodominant antigen recognized by HTLV-1-specific CTLs is the Tax protein particularly in association with HLA-A*0201 (Jacobson et al., 1990). These virus-specific CTLs, infiltrating CNS lesions, produce proinflammatory cytokines and show degranulation activity (Kubota et al., 1998; Yamano et al., 2002; Abdelbary et al., 2011). The frequency of Tax specific CD8^+^ T-cells is also higher in CSF than in PBMCs and is proportional to HTLV-1 PVL (Greten et al., 1998; Nagai et al., 2001). Tax-specific CTLs were also detected in spinal cord parenchyma (Matsuura et al., 2015). Strong Tax-specific CTL response has been considered to control the viral replication and play key role in developing of HAM/TSP. While Tax expression is generally suppressed in infected cells *in vivo* (Hanon et al., 2000b), CTL response to Tax is chronically activated, implying frequent exposure to newly-synthesized Tax protein *in vivo* (Rende et al., 2011). Single cell analysis demonstrated that Tax protein is expressed in intermittent but intense bursts (Billman et al., 2017; Mahgoub et al., 2018). Recently, exosome containing Tax was detected in virus-free CSF of patients with HAM/TSP and may therefore also be the target of CTLs (Anderson et al., 2018). These mechanisms may help maintain persistently activated CTL responses to HTLV-1. HBZ is also an immunogenic protein recognized by HBZ-specific CTL clones and HBZ-specific CTLs are detected in ATLL or HAM/TSP patients and ACs (Suemori et al., 2009; Macnamara et al., 2010). HTLV-1-infected individuals with HLA class I alleles strong binding the HBZ and the HBZ-specific CTL response was shown to be associated with a lower proviral load and a reduced risk of HAM/TSP (Macnamara et al., 2010; Hilburn et al., 2011). However, compared with Tax-specific CTLs, HBZ-specific CTLs are present at lower frequency in the peripheral blood and kill fewer HTLV-1-infected cells *in vitro* (Macnamara et al., 2010; Rowan et al., 2014). While HBZ is continuously expressed in HTLV-1 infected cells *in vivo*, the immunogenicity of HBZ is lower compared to tax, indicating that HTLV-1 could escape from host immune response and persistence of viral latency. Therapeutically, enhancing anti-HBZ immune responses or identifying HBZ epitopes that can induce a stronger CTL response in HAM/TSP may have clinical benefit.

### T Helper Cells

CD4^+^ helper T-cells are necessary for both CTL response and B cell reactivity against HTLV-1. The inflammatory cells in the CNS in early stages of HAM/TSP are dominated by CD4^+^ T-cells with relatively high proviral loads and elevated Tax and IFN-γ expression (Umehara et al., 1993; Furuya et al., 1997; Moritoyo et al., 1999). The most common HTLV-1 antigen recognized by CD4^+^ T-cells is the Env protein (Kitze et al., 1998; Goon et al., 2004). The frequency of HTLV-1 specific CD4^+^ T-cells were higher in patients with HAM/TSP than in ACs with a similar PVL (Goon et al., 2004; Nose et al., 2007). Additionally, the antiviral Th1 phenotype is also dominant among HTLV-1-specific CD4^+^ T-cells in patients with HAM/TSP (Goon et al., 2002), and higher frequency of IFN-γ, TNF-α, and IL-2 secreting CD4^+^ T-cells has been demonstrated in patients with HAM/TSP compared to ACs with a similar PVL (Goon et al., 2002, 2003). HTLV-1-infected CD4^+^ T-cells secrete IFN-γ (Hanon et al., 2000b; Araya et al., 2014), and the frequency of IFN-γ-secreting HTLV-1-specific CD4^+^ T-cells has been shown to be higher in patients with HAM/TSP than in asymptomatic HTLV-1 carriers with a similar proviral load (Goon et al., 2002). Immunogenetic analysis also demonstrates that possession of HLA-DRB1*0101 is associated with susceptibility to HAM/TSP in Japan (Jeffery et al., 1999). It has been demonstrated that HTLV-1 infected T-cells which coexpressed the Th1 marker CXCR3 and produced T-bet and IFN-γ were present in the CNS (Araya et al., 2014). When stimulated by IFN-γ, the astrocytes produce the CXCL10, which recruits more CXCR3^+^ T-cells, including more infected cell, to the CNS (Ando et al., 2013). These cells also produce pro-inflammatory cytokines such as IFN-γ, which stimulates astrocytes, further creating an inflammatory positive feedback loop with subsequent tissue damage in the CNS (Yamano and Coler-Reilly, 2017).

### Regulatory T-Cells

HTLV-1 preferentially and continuously infects CD4^+^CD25^+^ T lymphocytes *in vivo* (Yamano et al., 2005). CD4^+^CD25^+^ T-cells, termed Tregs, contribute to the maintenance of immunologic self-tolerance by inhibiting the activation and proliferation of self-reactive lymphocytes (Sakaguchi et al., 2001). In HAM/TSP patients, CD4^+^CD25^+^ T-cells contain higher amount of HTLV-1 PVL and levels of HTLV-1 *tax* mRNA expression than in CD4^+^CD25^−^ cells and induces various cytokines including INF-γ (Yamano et al., 2004). HTLV-1-infected CD4^+^CD25^+^ T-cells were not functionally suppressive but rather were shown to stimulate and expand HTLV-1 Tax-specific CD8^+^ T-cells (Yamano et al., 2004). In HAM/TPS patients, the levels of the forkhead box P3 (FoxP3) expression was decrease in CD4^+^CD25^+^ T-cells compared to ACs and healthy controls (Yamano et al., 2005; Oh et al., 2006). Furthermore, transduction of Tax reduced the *FoxP3* mRNA expression and inhibited the suppressive function of the CD4^+^CD25^+^ T-cells isolated from healthy donors (Yamano et al., 2005). HTLV-1 preferentially infects cells expressing the C-C chemokine receptor 4 (CCR4) ^+^CD4^+^ T-cells through the CCR4 ligand (CC chemokine ligand (CCL)22), which is produced by Tax (Hieshima et al., 2008). In HAM/TSP patients, the frequency of IFN-γ-producing CD4^+^CD25^+^CCR4^+^ T-cells was increased and correlated with disease activity and severity (Yamano et al., 2009). Anti-CCR4 monoclonal antibody effectively reduced the proviral load and proinflammatory cytokines in PBMCs from patients with HAM/TSP (Araya et al., 2014; Yamauchi et al., 2015). Clinical trial of anti-CCR4 monoclonal antibody has been performed and showed promising results (Sato et al., 2018a).

### Antibody Response

Robust humoral immune responses against HTLV-1 antigens have been reported (Osame et al., 1986; Kitze et al., 1996; Enose-Akahata et al., 2012), and it has been suggested that antibody syntheses might be related to both protective and pathogenic functions in HAM/TSP. Intrathecal antibody response to HTLV-1 inversely correlates with higher PVL and a worse prognosis (Puccioni-Sohler et al., 1999). HAM/TSP patients generally have a higher anti-HTLV-1 Ab titer than ACs with a similar HTLV-1 proviral load (Nagasato et al., 1991; Kira et al., 1992; Ishihara et al., 1994), and ATLL patients (Enose-Akahata et al., 2012). HTLV-1-Tax-specific antibody is elevated in HAM/TSP patients compared with both ACs and ATLL patients (Lal et al., 1994; Chen et al., 1997), and HTLV-1 specific antibody could distinguish HAM/TSP from ACs and ATLL (Enose-Akahata et al., 2012). However, the pathogenic role of these antibodies in HAM/TSP remains unclear. Antibodies against Tax and Gag p24, were reported to cross-react with host antigens, heterogeneous nuclear ribonucleoprotein A1 (hnRNPA1) and peroxiredosin-1 (PrX-1), respectively, suggesting a role for molecular mimicry in the autoimmune pathogenesis of HAM/TSP (Levin et al., 2002; Lee et al., 2008). However, subsequent analysis reported that the presence of hnRNPA1-specific antibodies in the CSF was not specific to this disease (Yukitake et al., 2008). Recent analysis shows intrathecal HTLV-1 specific antibody is significantly elevated in HAM/TSP patients compared to ACs, which is significantly correlated with antibody secreting B cells (ASCs) (Enose-Akahata et al., 2018). These results suggest that elevated ASCs may contribute to antibody production in the CSF and importance of the B cell compartment in the pathogenesis of developing of HAM/TSP. A more detailed analysis of B cell response and development including B cell/T-cell interactions will improve our understanding of the pathological mechanism of antibody secretion in HAM/TSP.

### Cytokine Expression

Elevated cytokine expression and production have been demonstrated in the peripheral blood and CNS lesion of patients with HAM/TSP (Jacobson, 2002). Inflammatory cytokines such as IL-1β, TNF-α, and IFN-γ were expressed by macrophages, astrocytes, and microglia in the active inflammatory lesions (Umehara et al., 1993). Tax has been shown to transactivate a number of the common γ chain family of cytokines and the receptors, such as IL-2/IL2R, IL-9, IL-15/IL-15R, and IL-21/IL-21R (Enose-Akahata et al., 2017). IL-2/IL-2R and IL-15/IL-15R cytokine loops, which induce the proliferation and the elevated cytolytic activity of CD8^+^ T-cells, are deregulated in HAM/TSP and contribute to a variety of immunological abnormalities characteristic of this disorder (Azimi et al., 1999). HTLV-1 Tax-specific CD8^+^ T-cells expressed high IL-15Rα (Azimi et al., 2001) and induced degranulation and IFN-γ expression when stimulated by IL-15 (Enose-Akahata et al., 2008). Elevated levels of IL-2, IL-9, and IL-15 driven by Tax lead to activate the Jak3/STAT5 pathway, and Jak3 inhibitor was shown to reduce *in vitro* immune activation in PBMCs from HAM/TSP patients (Ju et al., 2011). Monoclonal antibody therapy in HAM/TSP using a humanized antibody to IL-2 Rα showed reduction in the proviral load and spontaneous proliferation (Lehky et al., 1998), and a clinical trial of humanized-Mik-β-1, monoclonal antibody against IL-2/IL-15 Rβ, is currently underway, with promising early results (Waldmann, 2015). A selective inhibitor of IL-2 and IL-15 reduce the granulation and frequency of HTLV-1-specific CTLs (Massoud et al., 2015). The increase of the common γ chain family of cytokines and receptors may therefore be associated with the pathogenesis of HAM/TSP where anti-cytokine strategy maybe a promising therapeutic approach for HAM/TSP.

### Exosome

Exosomes are unilamellar extracellular vesicles originated from the endosomal compartment in various cell types (van der Pol et al., 2012). Several viruses take advantage of this mode of intracellular communication for incorporating viral components into exosomes (Anderson et al., 2016). Non cell-free virus has been detected in serum and CSF in HTLV-1-infected subjects (Pique and Jones, 2012). Exosomes derived from HTLV-1-infected cells appears to contain Tax protein and proinflammatory mediators as well as viral mRNA transcripts, including Tax, HBZ, and Env *in vitro* (Jaworski et al., 2014). Tax appears to be targeted for exosome entry by ubiquitination (Shembade and Harhaj, 2010; Jaworski et al., 2014), which was noted earlier to be an important target for endosomal sorting complex required for the transport pathway. Indeed, prior studies have shown Tax colocalized with organelles undergoing exocytosis (Alefantis et al., 2005a,b). Recent analysis showed that Tax protein was detected in exosomes isolated from CSF previously found to be negative for free HTLV-1 virus in HAM/TSP patients (Anderson et al., 2018). Exosomal Tax may therefore contribute to inflammatory cytokine production through packaging of these cytokines. Furthermore, HAM/TSP exosomes were shown to sensitize uninfected target cells for lysis by HTLV-1 specific CTLs (Anderson et al., 2018). HTLV-1 Tax in exosomes may therefore serve as a source of antigen that could be taken up by CNS resident cells to become targets for lysis by HTLV-1 Tax-specific CTLs. Exosomes may contribute to the continuous activation and inflammation observed in HAM/TSP and may be a potential biomarker that to be therapeutically targeted.

## Clinical Features

HAM/TSP is clinically characterized by chronic progressive spastic paraparesis, urinary incontinence and sensory disturbance. The mean age at onset is 43.8 years, and like many other autoimmune diseases, the frequency of cases of HAM/TSP is greater in women than in men (Nakagawa et al., 1995). Weakness of the lower limbs progresses to an abnormal spastic gait, and some patients may become wheelchair dependent or even bedridden. Hyperreflexia of the lower limbs is commonly seen, often accompanied by clonus and Babinski’s sign. Hyperreflexia of upper limbs is occasionally observed in some patients. The disease usually progresses slowly without remission; however, there is a subgroup of patients with rapid progression. The clinical course and rate of progression may vary greatly among patients. Older age at onset was associated with faster disease progression (Olindo et al., 2006; Nozuma et al., 2014). The median time from onset to require a unilateral walking aid was about 7 years, and wheel chair dependency was 18 years (Olindo et al., 2006; Coler-Reilly et al., 2016). Neurogenic bladder symptoms such as nocturia, frequency, and urgency are very common as well as constipation. It is also important to recognize that HAM/TSP patients have a risk to develop ATLL (Tamiya et al., 1995; Coler-Reilly et al., 2016).

## Diagnosis

The diagnosis of HAM/TSP is based upon a combination of characteristic clinical features and confirmation of HTLV-1 infection, along with exclusion of other disorders presenting spastic paraparesis (Yamano and Sato, 2012). The diagnostic criteria for HAM/TSP were initially proposed in 1989 (WHO, 1989). A modified diagnostic criterion is updated as levels of ascertainment as definite, probable, and possible with serological findings and/or detection of proviral DNA and exclusion of other disorders (De Castro-Costa et al., 2006). Recently, a new classification criterion based on disease activity has been proposed to help in providing adequate treatment for HAM/TSP (Sato et al., 2018b). The presence of HTLV-1 antibodies confirmed by western blot and/or a positive PCR should be demonstrated for confirmation of HTLV-1 infection. Recently digital droplet PCR (ddPCR) is used to quantify more precise PVL by determining absolute copy numbers instead of a standard curve in real-time PCR (Brunetto et al., 2014). Detection of anti-HTLV-1 antibodies in cerebrospinal fluid is necessary for the diagnosis of HAM/TSP. CSF examination revealed mild lymphocyte pleocytosis with mildly elevated protein and increased concentration of inflammatory markers such as neopterin, CXCL10, and CXCL9 (Sato et al., 2013). It is also necessary to exclude other disorders resembling HAM/TSP, such as multiple sclerosis (MS), neuromyelitis optica, and spinal cord compression. Neuroimaging is essential to exclude other diseases, but are not specific for HAM/TSP. Typical MRI showed spinal cord atrophy especially in the chronic phase without intensity changes. However, some patients with severe paraparesis and rapid progression show swelling of the spinal cord with T2 hyperintensity (Umehara et al., 2007). Recent advance of quantitative MRI techniques revealed that spinal cord cross-sectional areas correlated with clinical disability score, CD8^+^ T-cell frequency, and CSF PVL in HAM/TSP patients (Azodi et al., 2017). Reliable non-invasive biomarkers that correlate with the disease activity are clearly needed to inform appropriate treatment(s) for HAM/TSP.

## Treatment

Most therapeutic trials in HAM/TSP have aimed to suppress or modulate the immune response, or to reduce the HTLV-1 proviral load in an attempt to decrease the risk or modify the course of the disease. Corticosteroids are most commonly used to reduce the inflammation in the spinal cord, particularly in the early stage. Motor disability might be improved with steroids (Nakagawa et al., 1996; Croda et al., 2008), but improvement is usually not sustained and is difficult to tolerate due to various side effects of steroids. IFN-α is a therapeutic agent whose efficacy was demonstrated in randomized placebo-controlled trials (Izumo et al., 1996; Arimura et al., 2007). However, the therapeutic benefit is small. Reverse transcriptase inhibitors, which are used against HIV-1, were not effective against HTLV-1 in clinical trials (Taylor et al., 2006; Macchi et al., 2011). More recently, a humanized anti-CCR4 monoclonal antibody, mogamulizumab, effectively reduced both the PVL and inflammatory activity in cells obtained from patients with HAM/TSP (Yamauchi et al., 2015). Phase 1/2a clinical trial has demonstrated the safety and short-term effectiveness of mogamulizumab in patients with HAM/TSP (Sato et al., 2018a). Monoclonal antibody against IL-2 Rα resulted in reduction in the PVL and spontaneous proliferation (Lehky et al., 1998), and a clinical trial of humanized Mik-β-1, monoclonal antibody against IL-2/IL-15Rβ, is being evaluated in patients with HAM/TSP (Waldmann, 2015).

## Conclusion

Recent advances in research on HTLV-1 provide better understanding of the molecular pathogenesis and mechanisms of HAM/TSP, and several clinical trials of novel therapies for patients with HAM/TSP have been initiated. However, long-term improvement of motor disability and quality of life still have not been achieved in HAM/TSP patients, and the clinical management remains challenging. Given that HAM/TSP is characterized by activated T-cells in both the periphery and CNS, studies in HAM/TSP will be highly informative for clarifying the pathogenesis of other neuroinflammatory disorders such as multiple sclerosis. Novel approaches will be required to better define host-virus interactions and host immune response underlying the pathogenesis of HAM/TSP.

## Author Contributions

SN wrote the manuscript, and SJ supervised and contributed to the discussion and writing.

### Conflict of Interest Statement

The authors declare that the research was conducted in the absence of any commercial or financial relationships that could be construed as a potential conflict of interest.
